# Color doppler evaluation of cerebral-umbilical pulsatility ratio and its usefulness in the diagnosis of intrauterine growth retardation and prediction of adverse perinatal outcome

**DOI:** 10.4103/0971-3026.59747

**Published:** 2010-02

**Authors:** Shahina Bano, Vikas Chaudhary, Sanjay Pande, VL Mehta, AK Sharma

**Affiliations:** Department of Radiodiagnosis, Dr. Ram Manohar Lohia Hospital and PGIMER, New Delhi - 110 001, India; 1 Lady Hardinge Medical College and associated Hospitals, New Delhi - 110 001, India; 2 N.S.C.B. Medical College and Hospital, Jabalpur, Madhya Pradesh - 482 003, India

**Keywords:** Intrauterine growth retardation, pulsatility index, umbilical artery pulsatility index, middle cerebral artery pulsatility index, middle cerebral artery to umbilical artery pulsatility index ratio

## Abstract

**Objective:**

The aim of our study was to evaluate the usefulness of the pulsatility index (PI) of the umbilical artery (UA) and that of the fetal middle cerebral artery (MCA), as well as the ratio of the MCA PI to the UA PI (C/U ratio), in the diagnosis of small-for-gestational-age (SGA) fetuses and in the prediction of adverse perinatal outcome.

**Materials and Methods:**

The study population comprised 90 pregnancies of 30-41 weeks gestation that had been diagnosed clinically as intrauterine growth retardation (IUGR) over a period of 1 year. The UA PI and the MCA PI as well as the C/U ratio were calculated.

**Results:**

Of the 90 pregnancies in the study, 24 showed abnormal UA PI. Among these, 21 (87.5%) were SGA and 19 (79.2%) had adverse perinatal outcome. Of the four of the 90 pregnancies that showed abnormal MCA PI, all were SGA and had adverse perinatal outcome. Similarly, of the 20 out of 90 pregnancies that showed abnormal C/U ratio (<1.08), all 20 (100%) were SGA and had adverse perinatal outcome. The results were correlated with parameters of fetal outcome.

**Conclusion:**

Inferences drawn from the study were: (1) The C/U ratio is a better predictor of SGA fetuses and adverse perinatal outcome than the MCA PI or the UA PI used alone, (2) The UA PI can be used to identify IUGR *per se* and (3) The MCA PI alone is not a reliable indicator for predicting fetal distress.

## Introduction

Placental insufficiency, whether primary or secondary to maternal factors such as hypertension, poor nutrition, etc., is the most common cause of intrauterine growth retardation (IUGR), which is an important obstetric problem on account of the high associated perinatal mortality and morbidity. It is essential to recognize placental insufficiency early so that its hazards can be reduced, if not prevented.

Doppler USG enables a better understanding of the hemodynamic changes and has therefore become one of the most important clinical tools for fetomaternal surveillance in high-risk pregnancies. It can be credited with causing a significant decrease in perinatal mortality and morbidity.[1] The purpose of our study was to evaluate the usefulness of the pulsatility index (PI) of the umbilical artery (UA) and that of the middle cerebral artery (MCA), as well as the ratio of the MCA PI to the UA PI (C/U ratio), in the diagnosis of small-for-gestational-age (SGA) fetuses and the prediction of adverse perinatal outcome.

## Materials and Methods

The study population comprised 90 pregnancies of 30-41 weeks’ gestation that had been diagnosed clinically as IUGR and referred for USG over a period of 1 year.

Of the 90 cases, 45 showed normal fetal growth parameters, forming the control group; 45 fetuses had abdominal circumferences that were less than the 10^th^ percentile for their respective gestational ages along with elevated head circumference (HC)/abdominal circumference (AC) ratios, and these patients formed the study group. All the 90 patients were subjected to a repeat USG examination after 15 days, when the findings of the initial study were reconfirmed. All 90 patients were subjected to duplex Doppler examination, using a 3.5-MHz transducer with 3-mm sample volume and medium filter. During the examination, the patient was in a semirecumbent position and the fetus was in a quiet, resting state. The flow velocity waveforms were recorded from the UA and the fetal MCA. After technically satisfactory Doppler waveforms had been recorded, the PI of the UA and of the MCA was noted over three consecutive cardiac cycles and the ratio of the MCA PI to the UA PI (the C/U ratio) was calculated. A single reading was recorded in the control group and two readings were recorded in the study group. The pregnancies were followed-up and the final perinatal outcome of each case was noted. Various intrapartum and neonatal indicators were used to assess the outcome, with an adverse outcome being defined as the presence of one or more of these indicators. Gramellini *et al*.[2] have quoted the normal values of UA PI and MCA PI and the C/U ratio, and we used those values for interpreting the Doppler indices. The mean values quoted by Gramellini *et al*. ±2 SD were considered as normal for the corresponding gestational age. According to Gramellini *et al*.,[2] the C/U ratio remains constant in the last 10 weeks of pregnancy and therefore we used a single cut-off value of 1.08 throughout this study where all cases were of 30-41 weeks' gestation. Doppler velocimetry was considered as normal when the C/U ratio was above 1.08, and below that value, velocimetry was considered abnormal. The PI in the IUGR group was compared with that in the normal study group using the chi square test and Fischer's exact test. *P* < 0.05 was considered significant.

## Results

**The results are presented in the form of tables [Tables 1–5]** Of the 90 pregnancies in the study, 24 showed abnormal UA PI. Among these, 21 (87.5%) were SGA and 19 (79.2%) had adverse perinatal outcome. Four of the 90 pregnancies showed abnormal MCA PI and all four (100%) fetuses were SGA and all (100%) had adverse perinatal outcome. Perinatal death was noted in one pregnancy, which showed a normal MCA PI but an abnormal UA PI and C/U ratio. Twenty of the 90 pregnancies showed an abnormal C/U ratio (<1.08). Of these, 20 (100%) fetuses were SGA and all had an adverse perinatal outcome. Tables 4 and 5 depict the diagnostic performance of various Doppler flow indices in identifying SGA fetuses and predicting adverse perinatal outcome. The sensitivity and negative predictive values (NPVs) were higher for the C/U ratio and UA PI, whereas significantly lower for MCA PI. The specificity and positive predictive value (PPV) of the C/U ratio and MCA PI were equal to each other but higher than that of UA PI.

**Table 1:** Perinatal outcome of the study population according to the values of umbilical artery pulsatility index

Serial number	Normal PI (n = 66) (%)	Abnormal PI (n = 24) (%)	*P* value
Adverse perinatal outcome	5 (7.6)	19 (79.2)	<0.0001
Caesarian section for fetal distress	2 (3.0)	16 (66.7)	<0.0001
Apgar score <7 at 5 min	1 (1.5)	10 (14.7)	<0.0001
Stay in NICU >8 days	4 (6.1)	13 (44.2)	<0.0001
Still birth/perinatal death	-	2 (8.3)	<0.01
Small-for-gestational age (birth weight less than 10^th^ percentile for gestational age)	24 (36.4)	21 (87.5)	<0.0001

NICU - Neonatal intensive care unit; CS - Caesarian section

**Table 2:** Perinatal outcome of the study population according to the values of middle cerebral artery pulsatility index

Serial number	Normal PI (n = 86) (%)	Abnormal PI (n = 4) (%)	*P* value
Adverse perinatal outcome	20 (23.3)	4 (100)	<0.001
Caesarian section for fetal distress	15 (17.4)	3 (75.0)	<0.05
Apgar score <7 at 5 min	8 (9.3)	3 (75.0)	<0.01
Stay in NICU >8 days	14 (16.3)	3 (75.0)	<0.05
Still birth/perinatal death	1 (1.2)	1 (25.0)	<0.001
Small-for-gestational age (birth weight less than 10^th^ percentile for gestational age)	41 (47.7)	4 (100)	<0.05

**Table 3:** Perinatal outcome of the study population according to the cerebral-umbilical ratio

Serial number	Normal PI ratio (>1.08) (n = 70) (%)	Abnormal PI ratio (<1.08) (n = 20) (%)	*P* value
Adverse perinatal outcome	4 (5.7)	20 (100)	<0.0001
Caesarian section for fetal distress	1 (1.4)	17 (85.0)	<0.0001
Apgar score <7 at 5 min	2 (2.9)	9 (45.0)	<0.0001
Stay in NICU >8 days	4 (5.7)	13 (75.0)	<0.0001
Still birth/perinatal death	-	2 (10.0)	<0.01
Small-for-gestational age (birth weight less than 10^th^ percentile for gestational age)	25 (35.71)	20 (100)	<0.0001

**Table 4:** Diagnostic performance of pulsatility index and C/U ratio for small-for-gestational-age infants

n = 90	No. of findings				Sensitivity (%)	Specificity (%)	PPV (%)	NPV (%)	DA (%)
	
	TP	FN	FP	TN					
UA	21	24	3	42	46.7	93.3	87.5	63.6	70
MCA	4	41	0	45	8.9	100	100	52.3	54.4
C/U ratio	20	25	0	45	44.4	100	100	64.3	72.2

n - No. of study population; PPV - Positive predictive value; NPV - Negative predictive value; DA - Diagnostic accuracy; TP - True positive; FN - False negative; FP - False positive; TN - True negative

**Table 5:** Diagnostic performance of pulsatility index and C/U ratio for adverse perinatal outcome

n = 90	No. of findings				Sensitivity (%)	Specificity (%)	PPV	NPV	DA (%)
	
	TP	FN	FP	TN					
UA	19	5	5	61	79.2	92.4	79.2	92.4	88.9
MCA	4	20	0	66	16.7	100	100	76.7	77.8
C/U ratio	20	4	0	66	83.3	100	100	94.3	95.6

TP - True positive; FN - False negative; FP - False positive; TN - True negative

## Discussion

IUGR is a pathological condition strongly related to the development and function of the uteroplacental and fetoplacental circulations. An adequate fetal circulation is necessary for normal fetal growth. To facilitate this, remarkable changes occur in the maternal, placental and fetal vasculatures.

**Figure 1:**
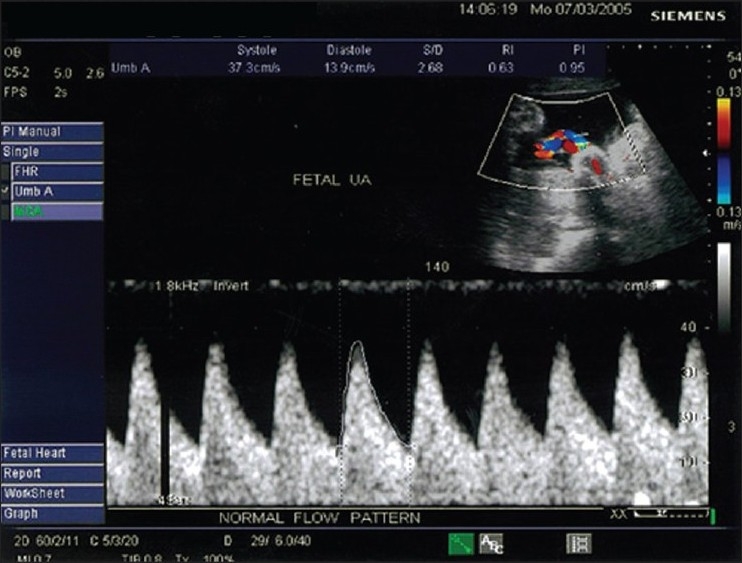
Color Doppler of the umbilical artery shows a normal UA waveform pattern with low impedance, high diastolic flow and decreased pulsatility index

**Figure 2:**
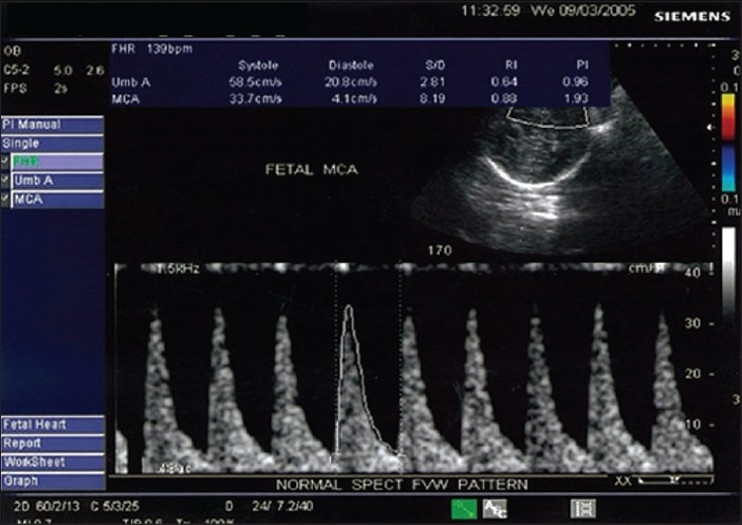
Color Doppler of the normal middle cerebral artery shows a normal MCA waveform pattern, with high resistance, low diastolic flow and increased pulsatility index

UA velocimetry correlates with hemodynamic changes in the fetoplacental circulation. With an increase in the number of tertiary stem villi and arterial channels, as the fetoplacental compartment develops, the impedance in the UA decreases. A diastolic component in the UA flow velocity waveform (FVW) appears during the early second trimester, i.e., at 15 weeks' gestation, and progressively increases with an increase in the gestational age. A mature UA FVW is usually achieved by 28- 30 weeks.[3] The normal UA waveform pattern shows low impedance and high diastolic flow with a low PI [Figure 1]. During normal pregnancy, the MCA shows high resistance and low diastolic flow with an increase in the PI index [Figure 2].

Gramellini *et al*.[2] calculated the C/U ratio and found that it remained constant in the last 10 weeks of pregnancy. We, therefore, used a single cut-off value of 1.08 for all cases of 30–41 weeks of gestation. Above this value, Doppler velocimetry was considered normal and, below it, abnormal. Using this cut-off value, we could divide the study population into two groups: Those with a normal ratio and those with an abnormal ratio.

**Figure 3 (a-c):**
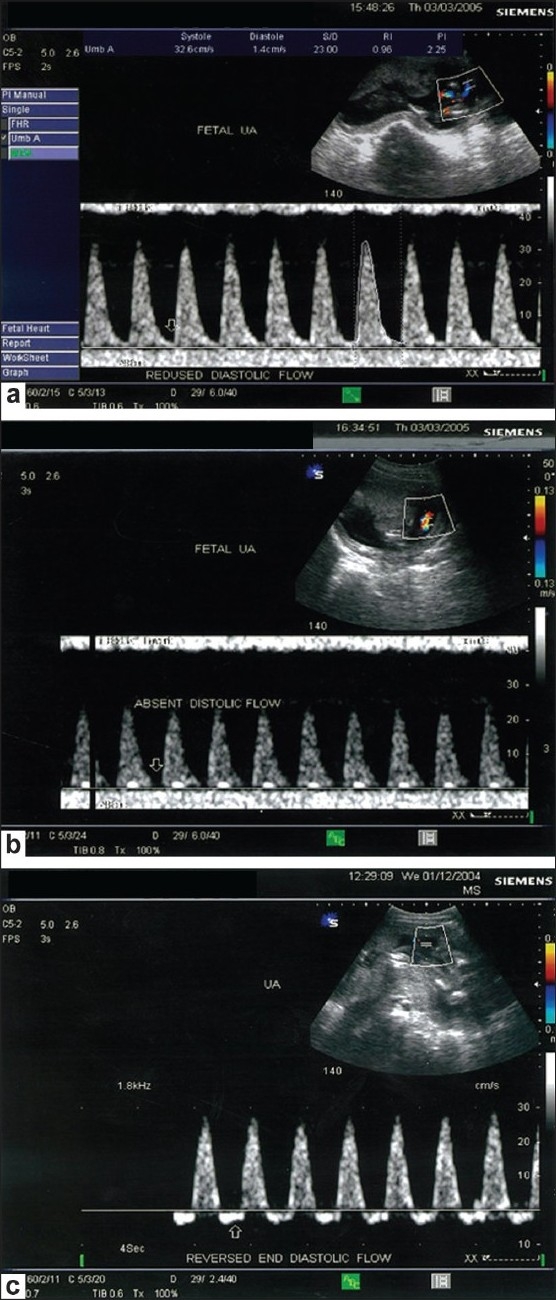
Abnormal umbilical artery waveform patterns showing markedly reduced diastolic flow and increased pulsatility index (a) absent diastolic flow (b) and reverse diastolic flow (c)

In IUGR, umbilical blood flow is significantly reduced, mainly due to changes in the placental vascular resistance. Giles *et al*.[4] have found that a decrease in the number of resistance vessels in the tertiary stem villi in the placenta causes an increase in resistance, leading to decreased flow through the UA and an increase in the UA PI. This is described as umbilical placental insufficiency. Fleischer and Schulman[1] have found that in IUGR complicated by pregnancy-induced hypertension, there is inadequate trophoblastic invasion of the spiral arteries, leading to increased resistance in the spiral arteries and decreased blood flow in the placental vascular bed and in the UA, thereby resulting in an increase in the UA PI. This is described as uteroplacental insufficiency. Several blood flow classes (0-IIIb) have been defined by Hofer *et al*.[5] to describe abnormal UA waveform patterns. Increasing pathological significance is ascribed to a decrease in diastolic flow (class II), absence of diastolic flow (class IIIa) and reversal of diastolic flow (class IIIb). All these patterns were associated with increased UA PI [Figure 3a–c]. Patients with absent end-diastolic volume (AEDV) and reverse end-diastolic volume (REDV) have the gravest outcome. Fetuses with AEDV require intensive surveillance as fetal well-being may deteriorate within a few days. Fetuses with REDV are most severely compromised. REDV is indicative of a preterminal fetal state.

**Figure 4:**
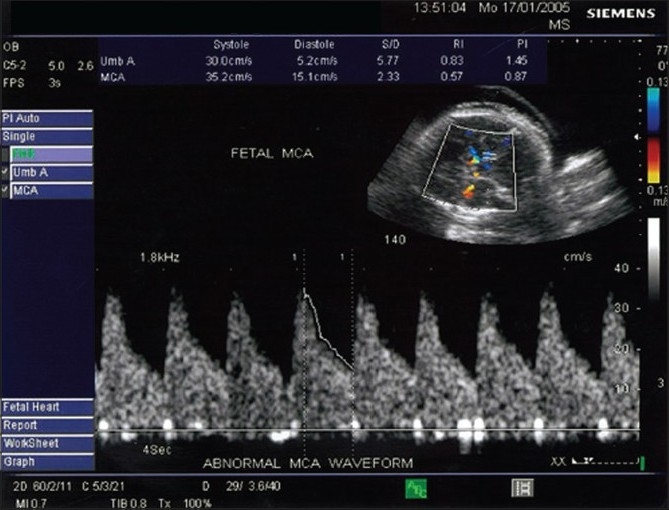
Abnormal middle cerebral artery waveform pattern shows low resistance and high diastolic flow due to cerebral vasodilatation (the brain-sparing effect)

In pregnancies with chronic fetal hypoxia, the blood volume in the fetal circulation is redistributed in favor of vitally important organs, i.e., the heart, kidneys and brain. Vasodilatation of the MCA, with an increase in diastolic flow through it, results in a decrease in its PI. The resulting hyperperfusion is considered pathological [Figure 4]. This ‘brain-sparing effect’ is associated with an abnormal C/U ratio (<1.08). However, if hypoxia persists, the diastolic flow returns to the normal level. Presumably, this reflects a terminal decompensation in the setting of acidemia or brain edema.[5]

Thus, in asymmetrical growth retardation, there is a high UA PI and low MCA PI. As a result, the C/U ratio is lower than normal in growth-retarded fetuses. A significant association between the C/U ratio and the HC/AC ratio can be seen. The C/U ratio remains constant during the last 10 weeks of gestation and provides better diagnostic accuracy than either vessels' PI considered alone.[2]

In our study, we found that the C/U ratio was a better predictor of SGA newborns and adverse perinatal outcome than either the MCA PI or UA PI alone. The C/U ratio demonstrated a 100% specificity and PPV in diagnosing IUGR and predicting adverse perinatal outcome, but had a low sensitivity of 44.4% and an NPV of 64.3% in diagnosing IUGR, but a relatively higher sensitivity of 83.3% and an NPV of 94.3% for predicting adverse perinatal outcome. The sensitivity and NPV of the C/U ratio were comparable to those of UA PI, but much higher than those of MCA PI.

Although the specificity and PPV of MCA PI was 100% in our study, the sensitivity and NPV were much lower. Therefore, a normal MCA PI may not be an indicator of fetal well-being. We also observed perinatal death in one pregnancy, which had shown a change in the MCA PI from abnormal to normal. This suggests that normalization of the MCA PI during chronic hypoxia may not be an indicator of fetal well-being as, in severe fetal hypoxia, the brain-sparing effect breaks down due to acidemia or brain edema and the low MCA PI becomes normal. Therefore, MCA PI alone is not a reliable indicator and, in such a situation, the increase in the UA PI can predict a severely growth-restricted infant. Detection of this change from abnormal to normal MCA PI with prolonged hypoxia is associated with severe growth retardation and predicts perinatal death.

We also found that for the diagnosis of IUGR, the sensitivity of UA PI was 46.7% as compared with 8.9% for MCA PI and 44.4% for the C/U ratio. This relatively higher sensitivity of the UA PI is probably because it directly reflects the resistance in the placental vascular bed. Thus, in cases of suspected IUGR, the measurement of PI value in the UA may be enough to detect IUGR.

Our results were more encouraging for the prediction of adverse perinatal outcome rather than diagnosing IUGR. It has been estimated that 41-86% of SGA babies can be detected with the routine use of symphysis-fundal height measurements.[6] According to one meta-analysis of USG fetal biometry, AC and estimated fetal weight (EFW) are the best predictors of fetal weight below the 10^th^ percentile.[7] In high-risk populations, the sensitivity of using AC below the 10^th^ percentile is 73-95%, whereas with EFW, the sensitivity is 43-89%. In low-risk populations, the corresponding sensitivities are 48-64% for AC and 31-73% for EFW.[8] Biophysical profile is another method for detecting IUGR, with a sensitivity of 77.7%.[9]

We further found that when compared with other modalities, although Doppler velocimetry was relatively less sensitive for diagnosing SGA fetuses, because uteroplacental insufficiency is just one cause of IUGR, it proved to be very useful in predicting IUGR fetuses at risk for adverse perinatal morbidity and mortality. None of the other modalities has been as promising in detecting adverse perinatal outcome as Doppler velocimetry. Among the number of biophysical tests available to assess fetal well-being, the most common methods are amniotic fluid volume (AFV), biophysical profile scoring (BPS) and nonstress test (NST). A reduced AFV (either measured by maximum vertical pocket < 2 cm or a four-quadrant amniotic fluid index (AFI) < 5 cm poorly correlates with the actual AFV and does not accurately predict adverse perinatal outcome. A review of 18 studies indicated that an AFI < 5 cm was associated with an increased risk of Cesarean section for fetal distress (relative risk [RR] 2.2; 95% confidence interval [CI] 1.5-3.4) and Apgar score of < 7 at 5 min (RR 5.2; 95% CI 2.4-11.3), but not with neonatal acidosis.[10] Large observational studies have shown an association between reduced AFV and perinatal morbidity and mortality, but the predictive value is poor (< 10%) and, also, there is little evidence to support intervention with isolated oligohydramnios (with a normal UA Doppler).[11] Yoon *et al*.[12] attempted to compare the performance of BPS and UA Doppler velocimetry in the identification of fetal acidemia, hypoxemia and hypercarbia as determined by PH and gas analysis of fetal blood obtained by cordocentesis in 24 patients. Although they found a strong relationship between the degree of fetal acedemia and hypercarbia and the results of UA Doppler velocimetry and BPS, Doppler velocimetry proved to be a better explanatory variable for these outcomes than the BPS.[12] Another study was performed by Gonzalez *et al*.[13] to compare the efficacy of NST, BPS and abnormal Doppler findings in predicting adverse perinatal outcomes in IUGR. The PPVs of abnormal Doppler for respiratory distress syndrome and the composite of adverse outcomes were 36% and 42%, respectively. Of the testing modalities compared, only abnormal Doppler significantly predicted respiratory distress syndrome and the composite of adverse outcome. Hence, they concluded that in cases of IUGR, the presence of abnormal Doppler was the best predictor of adverse perinatal outcome.[13] Padmagirison Radhika *et al*.[14] conducted prospective antenatal fetal surveillance in 55 women to compare the efficacy of Doppler velocimetry and NST in predicting fetal compromise *in utero* in cases of severe pre-eclampsia or IUGR. There were 29 cases with abnormal Doppler and 20 cases with abnormal NST. In addition, Doppler abnormalities preceded NST changes with a lead time of 4.14 days and there were 10 perinatal deaths, six of which occurred in the group where both the tests were abnormal. They concluded that Doppler identifies fetal compromise earlier than NST. The lead time helps to plan delivery in preterm compromised pregnancies, resulting in better perinatal survival.[14]

## Conclusion

Thus, we conclude that for the diagnosis of IUGR alone, Doppler velocimetry is not sensitive enough and a normal study does not rule out IUGR, which should then be diagnosed using other means. If the study, however, is abnormal, the specificity and PPV are virtually 100%. The sensitivity and specificity of the C/U ratio to assess adverse perinatal outcome however are high. Among the Doppler indices, the C/U ratio is a better predictor of SGA fetuses and adverse perinatal outcome than either the UAPI or the MCA PI alone, with a high specificity and PPV. However, measurement of the UAPI (among all the Doppler indices) is enough to detect IUGR per se, probably because UAPI is a direct reflection of the resistance in the placental vascular bed. The MCA PI alone is not a reliable indicator, and its efficiency in predicting fetal distress is found to be lower than that of UAPI.
